# A Dynamic Stochastic Model for DNA Replication Initiation in Early Embryos

**DOI:** 10.1371/journal.pone.0002919

**Published:** 2008-08-06

**Authors:** Arach Goldar, Hélène Labit, Kathrin Marheineke, Olivier Hyrien

**Affiliations:** 1 Service de Biologie Intégrative et de Génétique Moléculaire, Commissariat à l'Énergie Atomique, Gif-sur-Yvette, France; 2 Ecole Normale Supérieure, CNRS UMR 8541, Paris, France; University of Massachusetts Medical School, United States of America

## Abstract

**Background:**

Eukaryotic cells seem unable to monitor replication completion during normal S phase, yet must ensure a reliable replication completion time. This is an acute problem in early *Xenopus* embryos since DNA replication origins are located and activated stochastically, leading to the random completion problem. DNA combing, kinetic modelling and other studies using *Xenopus* egg extracts have suggested that potential origins are much more abundant than actual initiation events and that the time-dependent rate of initiation, *I(t)*, markedly increases through S phase to ensure the rapid completion of unreplicated gaps and a narrow distribution of completion times. However, the molecular mechanism that underlies this increase has remained obscure.

**Methodology/Principal Findings:**

Using both previous and novel DNA combing data we have confirmed that *I(t)* increases through S phase but have also established that it progressively decreases before the end of S phase. To explore plausible biochemical scenarios that might explain these features, we have performed comparisons between numerical simulations and DNA combing data. Several simple models were tested: i) recycling of a limiting replication fork component from completed replicons; ii) time-dependent increase in origin efficiency; iii) time-dependent increase in availability of an initially limiting factor, e.g. by nuclear import. None of these potential mechanisms could on its own account for the data. We propose a model that combines time-dependent changes in availability of a replication factor and a fork-density dependent affinity of this factor for potential origins. This novel model quantitatively and robustly accounted for the observed changes in initiation rate and fork density.

**Conclusions/Significance:**

This work provides a refined temporal profile of replication initiation rates and a robust, dynamic model that quantitatively explains replication origin usage during early embryonic S phase. These results have significant implications for the organisation of replication origins in higher eukaryotes.

## Introduction

Eukaryotic cells must ensure the complete duplication of their genome in a predictable time [1]. Replication initiates from multiple origins that fire at different times in S phase [2]. DNA synthesis progresses at replication forks bidirectionally from each origin, and stops when two converging forks meet. The timely completion of DNA replication must involve a tight coordination of initiation and termination with replication fork progression. Failure of a single origin or stalling of a single fork may result in the persistence of unreplicated DNA until mitosis, causing devastating chromosome segregation errors and inviability of daughter cells [1]. Accumulating evidence suggests that cells are unable to monitor the completion of DNA replication during normal S phase [3]–[6]. Therefore, some other mechanism must guarantee that S phase never lasts too long.

The replication completion problem is particularly crucial in early embryos of the frog *Xenopus laevis*, which have a very brief S phase (20 min) and a stochastic mechanism of replication initiation [1], [7]. Since forks progress at a rate of ∼0.5 kb.min^−1^ [7]–[11] the two forks emanating from a single origin cannot replicate more than 20 kb of DNA in 20 min. Thus, none of the required >3.10^5^ initiation events can be more than 20 kb from its neighbours (and even less if origins fire late in S phase). However, replication origins lack any sequence specificity in *Xenopus* embryos [7], [8], [12]. A random spatial positioning of origins implies an exponential distribution of interorigin distances. Since the *mean* spacing of initiation events is ∼10 kb the probability that two consecutive origins are spaced by >20 kb would be e^−20/10^ = 0.135. Even with a mean spacing of 5 kb the probability would still be 0.018, a figure incompatible with replication completion in <20 min. This paradox is known as the “random completion” or “random gap” problem [1], [13], [14]. It is not peculiar to Xenopus as replication initiation shows a large degree of stochasticity in yeast [15], [16] and human cells [17].

The problem might be solved if new origins could be continuously laid down on unreplicated DNA during S phase. However, this idea conflicts with what is known about origin regulation [18], [19]. Before S phase, origins are “licensed” by the coordinated action of the origin recognition complex (ORC), Cdc6 and Cdt1 proteins, which load complexes of the MCM2-7 proteins on DNA, thus forming “prereplicative complexes” (pre-RCs). During S phase, pre-RCs are converted into replication forks through phosphorylation and recruitment of other factors by two protein kinases, CDK and DDK (Cyclin- and Dbf4-dependent kinases). MCM2-7 are displaced from origins as they initiate and they most likely provide helicase activity in front of the forks. Importantly, MCM2-7 are prevented from rebinding chromatin until past next mitosis, which ensures that no DNA is replicated more than once in a single cell cycle. Thus, replication forks can only be assembled at origins that were licensed before S phase.

Two models have been proposed to solve the random completion problem. In the “regular spacing” model [7], [20], [21], origins are limiting so they must be assembled at regular (not random) intervals, albeit with no regard to specific DNA sequences, and be activated with very high efficiency. The weakness of this model is that accidental failure of just one or two out of >3.10^5^ consecutive origins could be lethal. The “origin redundancy” model [1], [22], [23] instead suggests that potential origins are much more abundant than actual initiation events and are activated randomly, and that the probability of origin firing increases as S phase progresses to allow rapid completion of unreplicated gaps. Electron microscopy [22], DNA combing [11], [24], [25] and two other DNA fiber techniques [20] were used to study the distribution of replication eyes on single DNA molecules replicating in *Xenopus* egg extracts. It was found that i) initiation occurs throughout S phase; ii) eye-to-eye distances are not regularly distributed; iii) the time-dependent rate of initiation, *I(t)*, increases as S phase progresses. These data clearly favour the origin redundancy model. Consistent with origin redundancy, the number of MCM2-7 complexes that are loaded on chromatin during the licensing reaction far exceeds the actual number of initiation events [26], [27], and each of them appears able to support initiation at a distance from ORC [22], [23], [28].

**Figure 1:**
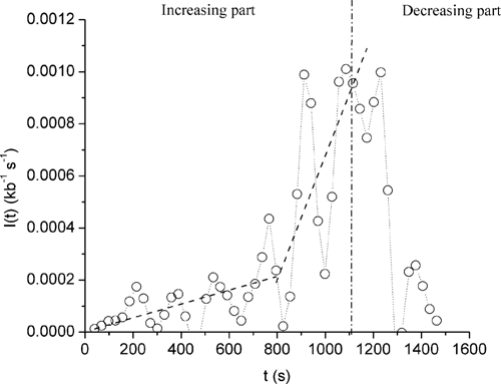
Replication initiation rate, *I(t)*, as a function of time. The open circles are the data points and the two dashed lines are linear fits presented in Figure 10 b in [31].

Given the complexity of this problem, the agreement of any model with the data must be assessed by quantitative analysis. The formal analogy between DNA replication and one-dimensional crystal nucleation (initiation), growth (elongation) and coalescence (termination), has allowed a mathematical analysis of the extensive datasets generated by DNA combing [29], [30]. In this model, all replication parameters (eye lengths, eye-to-eye distances, gap lengths, replicated fraction at time *t*) are derived from two fundamental parameters, replication fork velocity, (*v*), assumed to be constant through S phase, and the time-dependent rate of initiation, *I(t)*, assumed to be spatially homogeneous (i.e. potential origins are in large excess). Using an inversion procedure, a temporal profile of *I(t)* was extracted from the data [29], [31]. The extracted data show that *I(t)* markedly increases halfway through S phase then decreases sharply [31] (Fig 1). Using an analytical expression of *I(t)* that fits the increasing part of the data, the model could quantitatively account for the observed mean eye lengths, gap lengths and eye-to-eye distances at different times in S phase [29]. A formal study [32] demonstrated that initiating all origins at the beginning of S phase would lead to a broad ending time distribution, whereas an increasing *I(t)* narrows this distribution. To achieve a low completion failure rate, the experimentally observed *I(t)* requires a maximal fork density that is ∼3 times higher than the theoretical optimum but ∼6 times lower than if all origins were to fire at the onset of S phase. Therefore, both experimental and theoretical work support the origin redundancy model with a non-constant *I(t)*. However, the molecular mechanism that underlies the observed changes in *I(t)* remained obscure. Furthermore, these studies did not take into account the decreasing part of *I(t)* at the end of S phase, as the data were insufficient to rule out the possibility of large systematic errors at the end of S phase [30], [31].

In this article we have generated novel DNA combing data that allowed us to refine the temporal profile of *I(t)*. We confirm that I(t) increases through most of S phase but we also establish that it progressively decreases before the end of S phase. We have then used numerical simulations to explore plausible biochemical scenarios that might explain how the observed initiation function is produced. One possible explanation for the increase in initiation frequency is that once a certain number of forks have been established, further initiation depends on the recycling of some limiting component of the forks from completed replicons [1], [13], [29]. Efficient recycling would ensure a constant rate of DNA synthesis and thus a predictable replication completion time. Because the number of potential origins decreases as S phase progresses, efficient recycling would necessitate an increase in origin efficiency. Another possible mechanism is that the total amount of an initially limiting factor increases during S phase, due for example to its progressive nuclear import [33]. A third possible mechanism is that some feedback control modulates the frequency of initiation according to the density of already active forks [11], [34]. We show here that none of these mechanisms could on its own account for the data. We propose a novel model that combines time-dependent changes in availability of a replication factor and a fork-density dependent affinity of this factor for potential origins. This novel model quantitatively and robustly accounts for the observed changes in initiation rate and fork density through S phase. We discuss how the positive correlation between fork density and *I(t)* might be achieved and how this might also explain origin clusters and replication foci.

## Results

### Stochastic modeling of DNA replication

Our model genome consists of a lattice of *L* = 10^6^×100 bp blocs. Each bloc is represented by a 0 (0-block) if unreplicated or a 1 (1-block) if replicated. At the start of the computation, all blocks have a value of 0. Each 0-block is competent to initiate replication (i.e., potential origins are in vast excess). At each round of computation (equivalent to 10 seconds), a fraction of the 0-blocks initiate replication and are converted to 1-blocks. Replication forks are defined by the boundary between a 0- and a 1-block. At each round of computation, forks move by one block. Replication fork velocity (*v*) is therefore constant at *v* = 10 nucleotides.s^−1^ (nt.s^−1^). Converging forks stop when they merge. Blocks with value 1 cannot rereplicate. Replication is finished when all blocks have a value of 1.

We envision here a simple model of initiation governed by the encounter of a particle with a 0-block to convert it to a 1-block with probability *P(t)*. Following productive initiation, the particle is split in two halves, which remain associated with the two diverging forks. The merging of two converging forks regenerates a particle from two halves. The regenerated particle is released from the DNA and made available again for initiation. Thus, the total number of particles is equal to the sum of free and bound particles [*N_T_(t)* = *N_F_(t)*+*N_B_(t)*], and the total number of forks is twice the number of bound particles.

We assume that the chromosome is initially surrounded by *N_0_* particles. At each computation round, the maximum number of 0-blocks that may productively interact with a particle is defined as L_u_(t)*P(t), where *L_u_(t)* is the number of 0-blocks at time t (length of unreplicated DNA). We define the total number of new initiations at each round of computation, *i_t_*, as follows: *i_t_* = L_u_(t)*P(t) if L_u_(t)*P(t)<*N_F_(t)*, else *i_t_* = *N_F_(t)*. The frequency of initiation (i.e. number of initiations per unit time per unit length of unreplicated DNA) is *I(t)* = *i_t_/L_u_(t)*. Therefore the variations of *I(t)* solely depend on *P(t)* and *N_F_(t)*. *P(t)* has the same dimensions as *I(t)*: number of events per unit time per unit length of unreplicated DNA.

In the above model, the total number of particles *N_T_(t)* and their probability of productive encounter with potential origins *P(t)* may vary with time. Indeed in Xenopus, replication factors are accumulated by active transport into the nucleus during S phase [33]. Furthermore, productive initiation is governed by phosphorylation of several replication factors by CDK and DDK [35]–[37]. These kinases modify the affinity with which initiation factors interact with replication origins. CDK activity probably increases during normal S phase, as mitosis is approaching [38]. The time-dependency of *N_T_* and *P* introduced here allows us to explore the potential of these molecular mechanisms to quantitatively account for the observed profile of *I(t)*.

### Stationary scenarios

**Figure 2:**
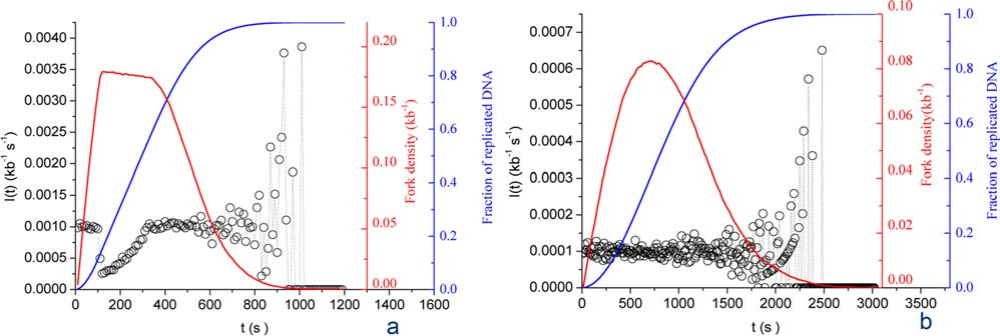
Computed *I(t)* for stationary scenarios. Open circles are numerical simulation data points. (A) Particle recycling scenario: *N_T_* = 10^4^;. *P* = 10^−3^ kb^−1^ s^−1^. (B) Particle abundance scenario: *N_T_* = 10^5^; *P* = 10^−4^ kb^−1^ s^−1^. Blue and red curves represent the simulated replicated fraction and the fork density, respectively.

In stationary scenarios, *N_T_* and *P* are held constant through S phase. We further define two subclasses, the “recycling” and the “abundance” scenario. In the “recycling” scenario, *P* is high enough that *N_T_* rapidly becomes rate limiting for initiation. Therefore, the frequency of initiation becomes limited by the release of the bound particles upon merge of active replicons, and the maximum density of forks approaches twice the total number of particles. Experimental data [1] indicate that the maximum density of forks is ∼1 per 5 kb (replicon size ∼10 kb), thus we set *N_T_* = 10^4^. If *P* is high enough to rapidly assemble a maximal number of forks (e.g. *P* = 10^−3^ kb^−1^ s^−1^), then *I(t)* behaves as shown on Figure 2a. After a brief initial phase where forks are assembled at a nearly constant rate, *I(t)* suddenly drops due to particle depletion to slowly reincrease as replicons merge. *I(t)* then stays relatively constant for most of S phase until small-number fluctuations appear due to the shrinking size of unreplicated DNA. This profile did not satisfyingly reflect the data (compare with Fig. 1), even after extensive parameter testing. Furthermore, this scenario results in a decrease in fork density during most of S phase, unlike the experimentally observed bell shape [11], [24], [25]. Therefore, no efficient recycling of limiting replication fork components occurs when *N_T_* is constant, even if *P* is high.

In the “abundance” scenario, *N_T_* is high enough that *P* is rate-limiting. Figure 2b shows the *I(t)* profile obtained with *N_T_* = 10^5^ and *P* = 10^−4^ kb^−1^ s^−1^. For most of the time *I(t)* = *P*, then the system runs into the final small-number fluctuations without showing the particle depletion phase observed in the previous scenario. Again, this does not adequately match the data (Figure 1). In conclusion, stationary scenarios cannot account for the observed *I(t)*, contrary to previous suggestions [1], [13], [29]. In the following sections, we explore what happens when either *N_T_* or *P* or both change with time.

### Increasing particle availability scenario

**Figure 3:**
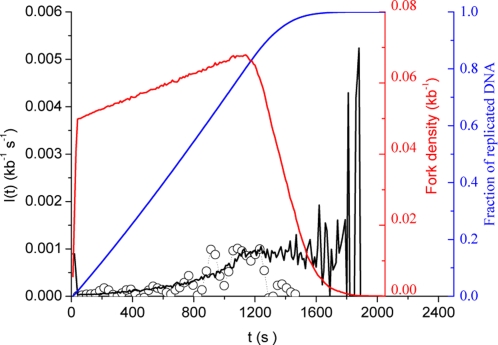
Fitting of the experimental *I(t)* (open circles) using an increased particle availability scenario. The solid black line is the best fit to the increasing part of the data using a Levenberg-Marquardt algorithm coupled with a dynamic Monte Carlo method (*N_0_* = 3100, *J* = 1 s^−1^ and *P* = 0.9×10^−4^ kb^−1^ s^−1^; *χ^2^* = 2.6×10^−8^). Blue and red curves represent the simulated replicated fraction and the fork density, respectively.

In this scenario, *N_T_* increases during S phase. Here we analyse the simple case where *N_T_* linearly increases with time at a defined rate *J* from an initial *N_0_* value. This scenario adequately mimics progressive nuclear import of a replication factor [33]. It can also reflect for instance mobilisation of a sequestered factor from an inactive nuclear pool [39]. Suitable values for the *N_0_*, *J* and *P* parameters were directly extracted by fitting this model to the data (Figure 3). One can see that for *N_0_* = 3100, *J* = 1 s^−1^ and *P* = 0.9×10^−3^ kb^−1^ s^−1^, a good fit to the increasing part of the data is obtained (Fig. 3; *χ^2^* = 2.6×10^−8^, calculated using a statistical weighting of each data point as described [40]). We found that this fit is as good or better as that obtained with two straight line segments [29], [31] (Fig. 1; *χ^2^* = 2.58×10^−8^) or with the previously proposed quadratic function *I(t)* = *I_2_t^2^* (where *I_2_* = 6.1 10^−10^ kb^−1^ s^−3^; *χ^2^* = 2.9×10^−7^) [32]. As for the recycling scenario (Fig. 2a), a transition from a *N_T_*-limited process during early S phase to a *P*-limited process later in S phase is observed. However, the increase of *N_F_(t)* now relies not only on an exponential release of particles from completed replicons, but also from their linear supply from an external source. Therefore, the shape of the increasing part of *I(t)* shows a linear to exponential transition, unlike the simple exponential increase in Fig. 2a, and the emergence of the plateau phase is delayed. Both factors account for the better agreement of the increasing particle availability scenario with the data. However, we note that in contrast to the increasing part of the data, the decreasing part remains unaccounted for.

### Increasing affinity scenario

**Figure 4:**
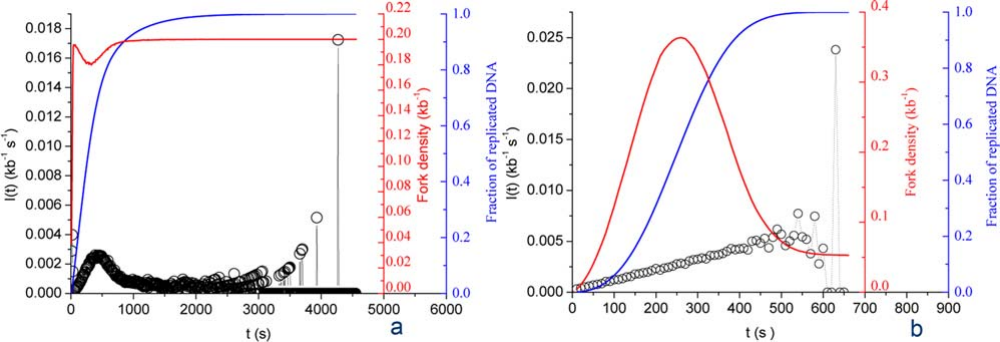
Computed *I(t)* for increased affinity scenarios. Open circles are numerical simulation data points. (A) Limiting particles scenario: *P(t)* = 10^−3^+10^−4^ *t*; *N_T_* = 10^4^. (B) Abundant particles scenario: *P(t)* = 10^−4^+10^−5^ *t*; *N_T_* = 10^5^. Blue and red curves represent the simulated replicated fraction and the fork density, respectively.

In this scenario, *P* increases during S phase while *N_T_* is kept constant. In other words, the probability that each particle is used for productive initiation increases during S phase. Here we analyse the simple case where *P* linearly increases with time at a defined rate *K* from an initial *P_0_* value (*P(t)* = *P_0_*+*Kt*). This hypothesis should not be taken to reflect any simple biological mechanism. The efficiency of origin usage probably depends in a complex manner on a number of molecular interactions whose affinity constants may change during S phase due to cell-cycle regulated phosphorylations and perhaps other modifications [19]. The proposed *P(t)* = *P_0_*+*Kt* was tried as a global model of these complex changes. No satisfactory fit to the data could be obtained, even after extensive trial. Let us first consider the case where particles are limiting: *P(t)* = 10^−3^+10^−4^ *t* and *N_T_* = 10^4^ (Figure 4a). After an initial burst of initiation, *I(t)* suddenly drops due to particle depletion, re-increases as replicons merge and declines again as the strong increase of *P(t)* results in a second phase of particle depletion. Compared with Figure 2a, the plateau is replaced by an extremum corresponding to an equilibrium between particle release and consumption and the small-number fluctuations are postponed till the very end of S phase. This profile does not adequately reflect the experimental data (Fig. 1), since the sharp increase of *I(t)* observed in mid-S phase is not reproduced. Furthermore, the fork density stays maximal throughout S phase, which is contrary to the experimental data. Let us now consider the case where particles are abundant: *P(t)* = 10^−4^+10^−5^ *t* and *N_T_* = 10^5^ (Figure 4b). A linear increase of *I(t)* is observed, reflecting the linear increase of *P(t)* and the lack of particle limitation. In conclusion, although the increased affinity scenario does not adequately account for the observed *I(t)*, it can suppress the plateau and defer the small-number fluctuations.

### Fork-density dependent affinity scenario

**Figure 5:**
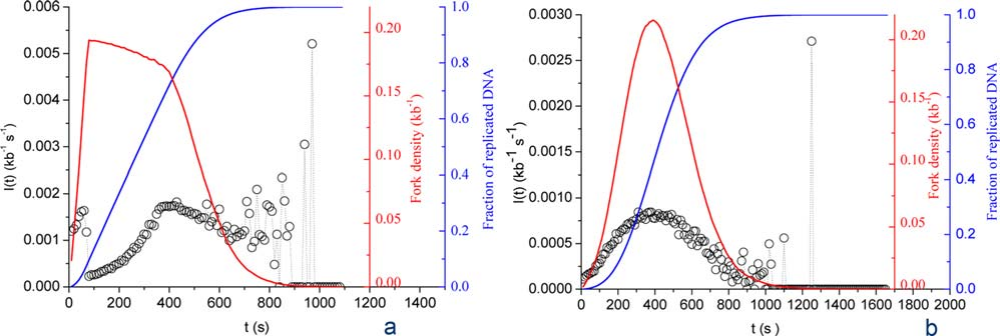
Computed *I(t)* for fork-density dependent affinity scenarios: *P(N_B_(t))* = *P_0_*+*P_1_*[1−exp(−*N_B_(t)/N_c_*)]. Open circles are numerical simulation data points. (A) Limiting particles scenario: *P_0_* = 10^−3^ kb^−1^ s^−1^, *P_1_* = 10^−3^ kb^−1^ s^−1^, *N_T_* = 10^4^ and *N_C_* = 7×10^3^. (B) Abundant particles scenario: *P_0_* = 10^−4^ kb^−1^ s^−1^, *P_1_* = 10^−3^ kb^−1^ s^−1^, *N_T_* = 10^5^ and *N_C_* = 7×10^3^. Blue and red curves represent the simulated replicated fraction and the fork density, respectively.

In the previous scenario, the chosen variation of *P(t)* was mathematically simple but lack obvious biological significance. Here we explore a distinct but related scenario, where *P(t)* is made dependent on fork density. Fork density is a reflection of both progression through S phase (and therefore of time) and consumption of replication factors. Therefore *P* is defined here as a function of *N_B_(t)*. As *I(t)* increases through most of S phase we need a feed-forward dependency of *P* on *N_B_(t)*. However, since the fork density reaches a plateau during mid-S phase, *P* has to satisfy a self-limiting law, which we define here as *P(N_B_(t))* = *P_0_*+*P_1_*[1−exp(−*N_B_(t)/N_c_*)]. This function has a minimum *P_min_* = *P_0_* for *N_B_* = 0 and a maximum *P_max_* = *P_0_*+*P_1_*. Here we set the value of *N_C_* so as to approximately match the experimentally observed maximum fork density (*N_C_* = 7×10^3^) [11]. Let us first explore the case where particles are limiting: *P_0_* = 10^−3^ kb^−1^ s^−1^, *P_1_* = 10^−3^ kb^−1^ s^−1^, *N_T_* = 10^4^ and *N_C_* = 7×10^3^ (Figure 5a). After a brief initial increase where fork assembly is governed by the increasing *P(t)*, *I(t)* suddenly drops when free particles are exhausted, to slowly reincrease as replicons merge. *I(t)* then reaches a maximum and drops to *I(t)* = 0 as merging of replicons becomes predominant. A few fluctuations are observed at the end of S phase. The main difference with Figure 2a is the disappearance of the mid-S phase plateau and the appearance of a decreasing part of *I(t)* at the end of S phase. However, no satisfactory fit to the experimental data could be obtained. Furthermore, this scenario results in a decrease in fork density during most of S phase, contrary to experimental observations.

Let us now explore the case where particles are abundant: *P_0_* = 10^−4^ kb^−1^ s^−1^, *P_1_* = 10^−3^ kb^−1^ s^−1^, *N_T_* = 10^5^ and *N_C_* = 7×10^3^ (Figure 5b). *I(t)* rapidly increases in an autocatalytical manner then decreases when merges become predominant. The end of S phase is marked by a prolonged lack of initiation. Again, this profile does not match the data, even after extensive parameter trial.

### Increasing particle availability combined with fork-dependent affinity

**Figure 6:**
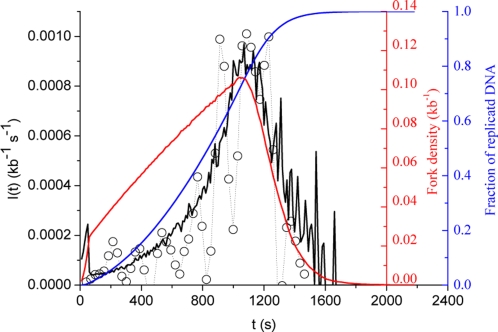
Fitting of the experimental *I(t)* (open circles) using a scenario that combines increased particle availability (*J*>0) and fork-density dependent affinity: *P(N_B_(t))* = *P_0_*+*P_1_*[1−exp(−*N_B_(t)/N_c_*)] and *N_C_* = 7×10^3^. The solid black line is the best fit to the increasing part of the data using a Levenberg-Marquardt algorithm coupled with a dynamic Monte Carlo method (*P_0_* = 10^−4^ kb^−1^ s^−1^, *P_1_* = 2×10^−3^ kb^−1^ s^−1^, *J* = 5 s^−1^and *N_0_* = 1000; *χ^2^* = 10^−9^). Blue and red curves represent the simulated replicated fraction and the fork density, respectively.

The increasing particle availability scenario on the one hand and the fork-dependent affinity scenarios on the other hand, have complementary strengths and weaknesses. The former better explains the increasing part of the data, the latter the decreasing part. In this section, we explore whether combining them can improve the fit to the data. This was indeed the case. The solid line on Figure 6 represents a fit to the data obtained by setting *N_C_* constant (*N_C_* = 7×10^3^) and adjusting the four free parameters (*P_0_* = 10^−4^ kb^−1^ s^−1^, *P_1_* = 2×10^−3^ kb^−1^ s^−1^, *J* = 5 s^−1^ and *N_0_* = 1000; *χ^2^* = 10^−9^). The fit shows an excellent agreement with both the increasing and decreasing part of the data. One might wonder whether the decrease in *I(t)* was due to a critical gap size below which initiation is prevented. Although plausible, this hypothesis appears unnecessary. The good fit to the decreasing part of the data, which contrasts with the simple increased availability scenario, was made possible just by making *P(t)* dependent on fork density.

We note that the model makes two predictions concerning the end of S phase. First, the previously neglected decreasing part of the data is biologically significant. Second, the very end of S phase is marked by a lack of initiations.

**Figure 7:**
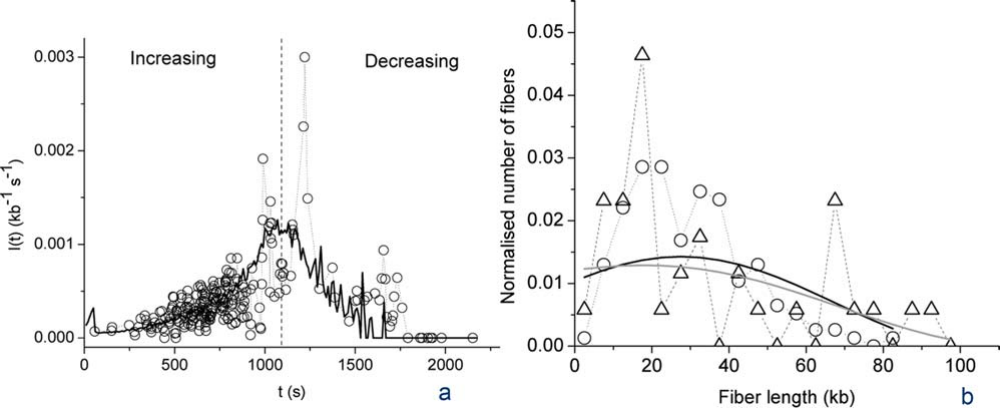
Analysis of a novel set of experimental data using the model shown on Figure 6. (A) Comparison of the novel data points (open circles, see text) with a rescaled fit to the previous data set (Figure 6). (B) Normalised distributions of fibre length in increasing (circles) and decreasing (triangles) parts of the data. The solid black and grey lines represent the smoothed distributions (using a 5 points Fourier filter) of the increasing and decreasing parts of the data, respectively.

To check these predictions and the robustness of the model, we analyzed a novel set of experimental data from which we directly extracted *I(t)* as explained in Materials and Methods. Sperm nuclei were replicated in *Xenopus* egg extract at a lower concentration than in the previous experiment (100 nuclei/µl instead of 1000 nuclei/µl) and origins were labeled by a brief pulse of digoxigenin-dUTP instead of the previous sequential labeling procedure. These experimental conditions have two advantages. The synchrony between individual nuclei is better, and small replication bubbles (0.5–2.0 kb) that were not considered significant with the previous labeling scheme can now be reliably scored, improving the determination of *I(t)*. *I(t)* was directly determined from the combed fibers based on the distribution of replication eyes smaller than 2 kb, that is, detectable initiation events that occured over a two-minute interval (see Material and Methods). The data are shown on Figure 7a (open circles). The statistical dispersion is significantly tighter than in Figure 1, and there are no negative values of *I(t)* which were caused by the numerical inversion procedure previously employed [29]. The general shape of *I(t)* is similar to Figure 1. Again, both an increasing and a decreasing part of the data are observed. Furthermore, the very late fiber replication stages are marked by a lack of initiation, as predicted by the model (solid line in Figure 6). The size distribution of early and late replicating DNA fibers is similar (Figure 7b), suggesting that the decreasing part of the data is not due to finite-size effects. The solid line on Figure 7a is a rescaling of the model shown on Figure 6 (no new fit was performed). The very good fit to the new data set suggests that the shape of *I(t)* is reproducible between experiments and invariant to rescaling.

**Figure 8:**
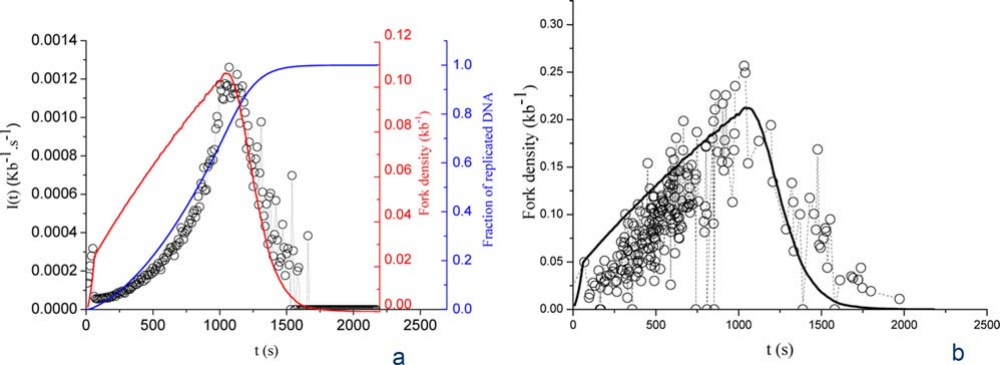
Temporal variation of fork density. (A) Comparison of the simulated fork density profile (solid red curve) and simulated *I(t)* (circles). Blue curve, simulated replicated fraction. (B) Comparison of the rescaled simulated fork density profile (solid black curve) and the experimentally determined fork density (circles).

Figure 8a shows the time-dependent changes in initiation (*I(t)*) and number of bound particles *N_B_(t)* (i.e. half the number of forks). During the first half of S phase, *I(t)* increases. *N_B_(t)* increases at a slower pace because of the progressive appearance of termination events. At mid-S phase *N_B_(t)* reaches a maximum, meaning that the frequency of termination becomes equal to the frequency of initiation. This sets the point at which *P(t)* and *I(t)* = P(t)*N_F_(t) start to decrease. Passing this point *N_B_(t)* decreases because termination predominates over initiation. Figure 8b compares the simulated fork density (*P_0_* = 10^−4^ kb^−1^ s^−1^, *P_1_* = 2×10^−3^ kb^−1^ s^−1^, *J* = 5 s^−1^ and *N_0_* = 1000 and *N_C_* = 7×10^3^) to the experimental data without further fit. A good agreement of the model with the experimental results is observed. Overall, these results provide good evidence for the robustness of the model.

## Discussion

**Figure 9:**
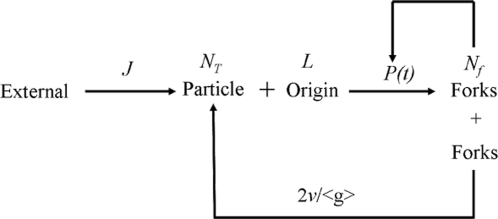
Model for regulation of replication initiation in *Xenopus* egg extracts. The bimolecular interaction of a trans-acting factor (particle) with an origin gives rise to initiation with a probability *P(t)* that depends on the density of already existing forks. The number (*N_T_*) of particles increases during S phase at a rate *J* from an initial *N_0_* value, due to recruitment by nuclear import or any analogous process. Initiation events result in a number of forks (*N_f_*) that merge at a frequency 2*v/<g>* (where *v* is the fork velocity and *<g>* the mean size of gaps at a given replication extent) and release particles that can be reused for initiation.

In this paper we provide a refined determination and analysis of the time-dependent frequency of replication initiation *I(t)* in *Xenopus* egg extracts. We confirm that *I(t)* increases through S phase but also establish that it smoothly decreases to 0 before the end of S phase. We have explored plausible scenarios to explain these features. Scenarios such as a simple recycling of a limiting replication fork component are clearly ruled out. On the contrary, a more elaborate model (Figure 9), featuring time-dependent changes in availability of a limiting replication factor and fork-density dependent changes in the affinity of this factor for potential origins, accurately described the data. This is the simplest model we found that accurately describes the data with a minimal set of parameters. The proposed model is certainly much simpler than the real biochemical mechanisms that regulate initiation. However, it formulates two testable hypotheses about the global processes that ensure replication completion, notwithstanding their biochemical details.

One prediction of this model is that a limiting component of replication forks accumulates during S phase. Biochemical experiments in *Xenopus* egg extracts have suggested that the binding of Cdc45 to chromatin is rate-liming for DNA replication [23]. Cdc45 is therefore a good candidate for the limiting “particle” described in our scenario as it is both a stable component of replication forks and is actively concentrated in the nucleus during S phase (J. Walter, pers. comm), but it is certainly not the only possible candidate [41]. It would be interesting to determine *I(t)* in the presence of controlled amounts of the candidate factors. The “accumulating factor” hypothesis predicts that if replication is stalled, e.g. by aphidicolin, the factor should accumulate, such that after release from the block origin firing should resume at an increased rate (provided that N_F_(t)≪L_u_(t)*P(t) at the start of the block). A previous experimental test failed to confirm this prediction [25], as the rate of origin firing was unchanged after aphidicolin release. However, it is quite possible that replication stalling activates a checkpoint that blocks accumulation of the limiting factor [42] or lowers *P*, so that the rate of initiation cannot increase following release.

A second prediction of the model is that the rate of origin firing is tied to the density of replication forks. It has been previously proposed that *Xenopus* egg extracts monitor the density of forks through an ATM/ATR-dependent checkpoint, which in turn downregulates the frequency of initiation [11], [34]. This proposal implies that *I(t)* is negatively correlated with fork density, which is not consistent with the data (although it may explain the self-limiting nature of the dependency of *P* on *N_B_(t)*). In contrast, our novel model implies a positive correlation between fork density and *I(t)*. It has been shown that Cdc45 recruits Cdk2 to replication foci to facilitate chromatin decondensation during S phase [43]. It is easy to imagine how the recruitment of Cdk2 in the vicinity of Cdc45-containing forks might facilitate origin activation.

Our model does not explain the occurence of synchronous origin clusters [11], [20], [21] but may be modified to do so by making the positive correlation between fork density and *I(t)* local rather than global. This may be justified if Cdk2 (or another global origin trigger) is recruited to forks by physical interaction with Cdc45 (or another stable fork component) and preferentially activates origins in the vicinity of existing forks.

Another limitation of our model is that it postulates a homogeneous intranuclear concentration of a limiting “particle” whereas the intranuclear concentration of many replication factors shows inhomogeneities known as replication foci [44]. The fork-promoted initiation of novel forks may contribute to this local accumulation of replication factors, in addition to the partial synchrony of adjacent origins.

A further limitation of this study is that all the models explored are based on recycling of a replication-fork factor. It is certainly conceivable that other mechanisms independent of recycling could fit the data equally well. However, the simplicity of our model, and the robustness of the fit to the data leads us to hope that the model captures some of the logic of S phase. The model makes sense regarding the necessarily limited amount of resources required to assemble replication forks and the desired autonomy and robustness of the mechanisms that control orderly progression through S phase and timely completion of DNA replication. The general nature of the proposed model pushes us to explore, in a future work, whether these findings can be generalized to other eukaryotes. It will also be interesting to see whether this model predicts the pattern of origin firing following perturbation of replication fork progression and can be modified to incorporate the effects of checkpoint mechanisms influencing origin firing.

## Materials and Methods

All simulations were performed using a dynamic Monte-Carlo method using a Matlab platform. The source code can be obtained upon request.

The data shown on Figure 7 were obtained as follows. *Xenopus* sperm nuclei were incubated in *Xenopus* egg extracts (100 nuclei/µl) as described [11] in the presence of 20 µM digoxigenin-dUTP (Roche Applied Sciences) added at *t* = 0. Reactions were chased at 20 and 25 min with 2 mM dTTP and stopped at *t* = 120 min by dilution with ice-cold PBS and processed for combing and detection of the digoxigenin label as described [11]. The combed DNA fibres were sorted according to replication extent *f*. For each fiber, eyes 0.5–2.0 kb (detectable initiation events that occured over a two-minute interval) were counted and the number was divided by 2 min and by the length of unreplicated DNA, in order to derive the frequency of initiation as a function of *f*. The *I(f)* data points were converted to *I(t)* using an *f(t)* curve determined from kinetic measurements of combed DNA. The fork density (Fig. 8b) was obtained by counting the number of forks and dividing by the length of each combed DNA fibre. The combed DNA fibres were sorted according to their replication extent *f* and the latter was converted to time using an *f(t)* curve determined as above.

This work and previous modelling of DNA combing data [29] rely on the assumption that DNA fibers of comparable replication extents have similar replication starting times. This allows to replot the frequency of initiation and the density of forks as a function of time rather than as a function of local replication extent. However, individual nuclei in a replication reaction enter S phase at slightly different times, and origins are organized as weakly synchronous clusters that fire at different times in a single nucleus [11], [20], [21]. Therefore, the time origin in the data plots more exactly reflects each fiber's starting time than the start of S phase. It should be noted that the transformation from *f* to *t* is sensitive to fiber length. However we do not believe that the finite size of our fibers biases this transformation because their size distribution is identical for early and late fibers (Figure 7b).
